# Supplementary material for: Identification of photoperiod-induced specific miRNAs in the adrenal glands of Sunite sheep (Ovis aries)
Source: Front Vet Sci. 2022 Jul 22;9:888207. doi: 10.3389/fvets.2022.888207 (PMC9354845; doi:10.3389/fvets.2022.888207)
Supplement: Supplementary file 1 [file Data_Sheet_1.ZIP › Supplementary file/miRDeep2 file.zip!/miRDeep2 file/Know_Structure/LP42A2/oar-mir-10b.pdf]

miRBase precursor : oar-mir-10b
Total read count : 677596
oar-miR-10b read count : 675583
remaining reads : 2013

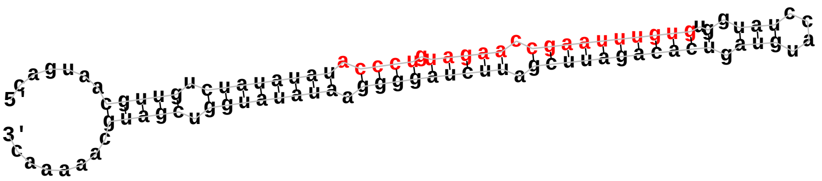

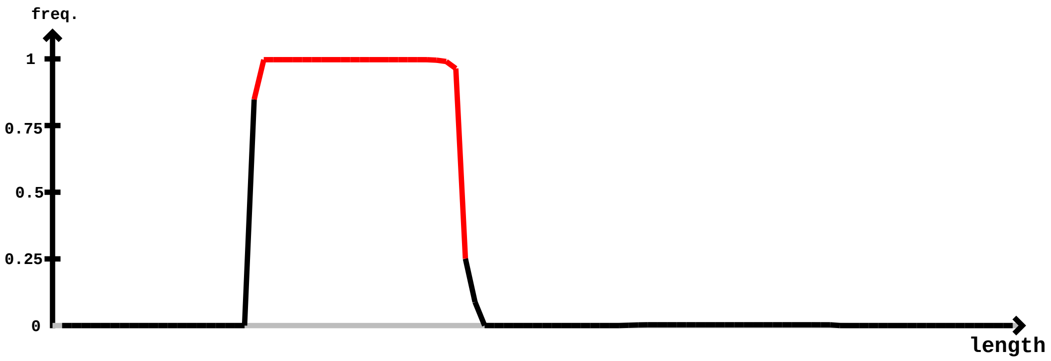


```
                                oar-miR-10b
5'- caguaacguugucuauauauacccuguagaaccgaauuugugugguauccauguagucacagauucgauucuaggggaauauauggucgaugcaaaaac   -3'    exp
    ......(((((.((((((((.((((.(((((.((((((((((.(.(((....))).))))))))))).)))))))))).)))))))).))))).......      reads      mm         sample
    caguaacguugucuauauau...............................................................................      3          0          seq
    .................uauacccuguagaaccgaauuugu..........................................................      1          0          seq
    ..................auacccuguagaaccgaauuug...........................................................      2          0          seq
    ..................auacccuguagaaccgaauuugu..........................................................      12         0          seq
    ..................auacccuguagaaccgaauuugug.........................................................      1          0          seq
    ...................uacccuguagaaccgaa...............................................................      14         0          seq
    ...................uacccuguagaaccgaau..............................................................      101        0          seq
    ...................uacccuguagaaccgaauu.............................................................      1076       0          seq
    ...................uacccuguagaaccgaauuu............................................................      2857       0          seq
    ...................uacccuguagaaccgaauuug...........................................................      16311      0          seq
    ...................uacccuguagaaccgaauuugu..........................................................      452089     0          seq
    ...................uacccuguagaaccgaauuugug.........................................................      78420      0          seq
    ...................uacccuguagaaccgaauuugugu........................................................      23673      0          seq
    ...................uacccuguagaaccgaauuugugug.......................................................      33         0          seq
    ...................uacccuguagaaccgaauuugugugg......................................................      2          0          seq
    ....................acccuguagaaccgaau..............................................................      5          0          seq
    ....................acccuguagaaccgaauu.............................................................      111        0          seq
    ....................acccuguagaaccgaauuu............................................................      456        0          seq
    ....................acccuguagaaccgaauuug...........................................................      1475       0          seq
    ....................acccuguagaaccgaauuugu..........................................................      31267      0          seq
    ....................acccuguagaaccgaauuugug.........................................................      31577      0          seq
    ....................acccuguagaaccgaauuugugu........................................................      35923      0          seq
    ....................acccuguagaaccgaauuugugug.......................................................      96         0          seq
    ....................acccuguagaaccgaauuugugugg......................................................      5          0          seq
    ....................acccuguagaaccgaauuuguguggu.....................................................      4          0          seq
    .....................cccuguagaaccgaauuugu..........................................................      7          0          seq
    .....................cccuguagaaccgaauuugug.........................................................      10         0          seq
    .....................cccuguagaaccgaauuugugu........................................................      31         0          seq
    ......................ccuguagaaccgaauuugu..........................................................      3          0          seq
    ......................ccuguagaaccgaauuugug.........................................................      7          0          seq
    ......................ccuguagaaccgaauuugugu........................................................      7          0          seq
    .......................cuguagaaccgaauuugu..........................................................      4          0          seq
    .......................cuguagaaccgaauuugug.........................................................      1          0          seq
    .......................cuguagaaccgaauuugugu........................................................      2          0          seq
    ........................uguagaaccgaauuugu..........................................................      1          0          seq
```

oar-miR-10b

```
caguaacguugucuauauauacccuguagaaccgaauuuguguugguauccauguagucacagauucgauucuaggggaauauauggucgaugcaaaaac
........................................................ucacagauucgauucuagggga.....................    1    0    seq
........................................................ucacagauucgauucuaggggaa....................    1    0    seq
.........................................................cacagauucgauucuagggg......................    1    0    seq
.........................................................cacagauucgauucuaggggaa....................    5    0    seq
.........................................................cacagauucgauucuaggggaau...................    6    0    seq
..........................................................acagauucgauucuagg........................    1    0    seq
..........................................................acagauucgauucuagggga.....................    32   0    seq
..........................................................acagauucgauucuaggggaa....................    45   0    seq
..........................................................acagauucgauucuaggggaau...................    701  0    seq
..........................................................acagauucgauucuaggggaaua..................    1    0    seq
...........................................................cagauucgauucuaggg.......................    1    0    seq
...........................................................cagauucgauucuagggga.....................    10   0    seq
...........................................................cagauucgauucuaggggaa....................    16   0    seq
...........................................................cagauucgauucuaggggaau...................    796  0    seq
...........................................................cagauucgauucuaggggaaua..................    8    0    seq
...........................................................cagauucgauucuaggggaauau.................    1    0    seq
............................................................agauucgauucuagggga.....................    7    0    seq
............................................................agauucgauucuaggggaa....................    5    0    seq
............................................................agauucgauucuaggggaau...................    340  0    seq
............................................................agauucgauucuaggggaaua..................    17   0    seq
............................................................agauucgauucuaggggaauau.................    12   0    seq
............................................................agauucgauucuaggggaauaua................    2    0    seq
```
